# The Physiological Antagonism of Remedies

**Published:** 1881-03

**Authors:** 


THE PHYSIOLOGICAL ANTAGONISM OF REMEDIES.—We regret very much that limited space prevents our giving, at least, a synopsis of the able and valuable lectures of Professor Bartholow, delivered before the Alumni Association of the College of Physicians and Surgeons, New York, and known as the Cartwright Lectures. But we will endeavor to place the leading facts connected with the action of the more prominent agents, in such a manner as to show their antagonisms. Before proceeding to do so, however, we will quote a paragraph on what Dr. Bartholow says of *Physiological Antagonism:*

"By physiological antagonism, is meant a balance of opposed actions on particular organs or tissues. As disease is a pathological physiology, so far, at least, as relates to function, the derangements produced by disease may be opposed by other derangements set up by medicinal substances. The antagonism, or opposition of actions, may extend through the whole range of effects, or it may be limited to a few points. Indeed, some of the most valuable instances of antagonism are thus limited, and there are few, if any, examples of antagonism, in which the opposition of actions is universal. In popular medical opinion, by the term physiological antagonism, is meant an opposition of action of poisonous medicinal agents, in that the effects of the one may be exactly counter-balanced by the effects of the other. According to this conception of the subject, when a letheal dose of one agent is administered, the effects may be removed by an opposing agent so given as to produce exactly opposite effects; therefore, the poisonous action ceases, because in the whole range of the effects of the two agents, they are exactly antagonized.

"This conception of physiological antagonism is exaggeration; for such completeness of opposing action is as rare as exact similitude in remedies acting in the same way." The following arrangement will present some of the most prominent articles, which antagonize in their physiological action upon the system, and we will tabulate them thus, viz:—

| PRO. | CONTRA. |
|---|---|
| Opium. | Belladonna. |
| Its preparations, as Morphia salts. | Its preparations as Atropia salt. |
| Laudanum, &c. | Extract Belladonna. |
| | Tincture Belladonna, &c. |
| Belladonna. | Opium. |
| Its preparations, as Atropia salts. | Its preparations, as Morphia salts. |
| Extract Belladonna, | Laudanum. |
| Tincture Belladonna, &c. | Paregoric, &c. |
| Atropia. | Muscarine. |
| " | Physostigma. |
| " | Pilocarpine. |
| Morphia Muriate. | Calabar Bean. |
| " " | Cafine. |
| " " | Thein. |
| Calabar Bean. | Strychnia. |
| Atropia. | Bromal Hydrate. |
| Aconite. | Digitalis. |
| Atropia. | Hydrocyanic Acid. |
| Ammon Carb. | " " |
| Strychnia. | Woorari. |
| " | Hydrate Chloral. |
| " | Physostigma. |

| | |
|---|---|
| Veratrum Veride. | Opium and preparations. |
| " " | Alcohol. |
| Atropia. | Aconite. |
| " | Quinia. |
| Chloral. | Strychnia. |
| " | Picrotoxine. |
| " | Atropia. |
| Opium. | Veratrum Veride. |
| " | Gelsemium. |
| " | Aconite. |

The foregoing list embraces the bulk of *antagonistic* articles known to us at present. But the field contains a mine of wealth and the laborers embrace many thinking heads and willing hands in the foremost ranks of the profession, throughout Christendom ; and it is not expecting too much to look for a therapeusis founded upon known laws of cure, ere the close of the present century ! In a future issue of the Journal mention will be made of some of the antagonisms of remedies to disease.

